# The New Health Bill before Congress

**Published:** 1885-02-20

**Authors:** 


### THE NEW HEALTH BILL BEFORE CONGRESS.

The editor of the New Orleans Medical Journal says of the above measure:

The bill, while being amendatory to the act of March 3d, 1879, possesses so many distinctive features that its passage will result in an entire and radical change in the National Board of Health as it has existed for the past six years, creating a new Board under a new plan of organization. This feature, which makes the Board constituted of one member from each State Board of Health, is the strongest in its text, and one which alone should recommend its passage. A Federal Board of Health, so constituted, cannot but prove of immense and incalculable benefit to the country at large, and particularly to those States exposed to the possible introduction of infectious diseases through their ports of entry.

